# Diagnostic Ability of Transverse Axial Images Obtained by Optical Coherence Tomography for Detecting Anterior Displacement of Peripapillary Tissues in Papilledema

**DOI:** 10.1111/ceo.14561

**Published:** 2025-06-01

**Authors:** Gabriel Castilho S. Barbosa, Rodrigo S. Pegado, Fernanda N. Susanna, Kenzo Hokazono, Mário Luiz R. Monteiro, Leonardo Provetti Cunha

**Affiliations:** ^1^ Department of Ophthalmology and Otorhinolaryngology Faculdade de Medicina da Universidade de São Paulo São Paulo Brazil; ^2^ Department of Ophthalmology Cleveland Clinic Cole Eye Institute Cleveland Ohio USA; ^3^ Department of Ophthalmology Federal University of Paraná Curitiba Paraná Brazil; ^4^ Department of Ophthalmology Federal University of Juiz de Fora Juiz de Fora Minas Gerais Brazil; ^5^ Juiz de Fora Eye Hospital Juiz de Fora Minas Gerais Brazil

**Keywords:** anterior displacement of the optic disc, idiopathic intracranial hypertension, papilledema

## Abstract

**Background:**

To evaluate the diagnostic ability of optical coherence tomography (OCT) images to detect posterior pole deformation and anterior displacement of the optic disc (ADOD) in papilledema before and after intracranial pressure reduction with medical treatment. Additionally, we compared the analysis of these images with peripapillary retinal nerve fibre layer (pRNFL) thickness measurements in detecting papilledema.

**Methods:**

In this retrospective, observational, descriptive, and comparative study, participants underwent swept‐source OCT with high‐resolution imaging. Papilledema cases were analysed at baseline and 7–30 days post‐treatment. Two masked examiners independently assessed images for ADOD.

**Results:**

The study included 82 eyes from 41 patients, with 50 eyes having papilledema and 32 eyes with optic disc drusen (ODD) as controls. At baseline, ADOD was observed in 50% of papilledema eyes and 32% post‐treatment. A reduction in ADOD was noted in 34% of papilledema eyes. Cohen's *κ* coefficient indicated substantial agreement (0.799). Mean pRNFL thickness significantly decreased from baseline to post‐treatment (305.4 ± 140.2 vs. 245.3 ± 94.5 μm, *p* < 0.001). Logistic regression demonstrated an association between mean pRNFL thickness and ADOD at baseline (OR = 1.016, *p* = 0.001) and post‐treatment (OR = 1.012, *p* = 0.004).

**Conclusion:**

OCT B‐scan analysis is an effective tool for detecting ADOD and posterior pole deformation in papilledema and distinguishing it from ODD. ADOD correlates with the severity of optic disc oedema and may serve as a more sensitive or specific indicator of treatment response compared to pRNFL thickness. These findings highlight the value of OCT axial imaging in diagnosing and monitoring papilledema, offering clinicians a reliable method for assessing disease progression and treatment efficacy.

## 1 Introduction

Intracranial hypertension (IH) is a neurological condition marked by elevated cerebrospinal fluid (CSF) pressure, which may be idiopathic (IIH) or secondary due to various conditions such as trauma, haemorrhage, tumours or infections [1]. Regardless of its aetiology, increased intracranial pressure can lead to the development of papilledema, which manifests as optic disc oedema resulting from the transmission of elevated pressure through the subarachnoid space surrounding the optic nerve. In papilledema, visual acuity is often preserved in the early stages; however, if left untreated, it can progress to permanent vision loss due to chronic optic disc oedema and damage to the retinal ganglion cells [2, 3, 4, 5].

Papilledema serves as a pivotal clinical sign in the diagnosis of IH, regardless of its underlying cause, reflecting elevated CSF pressure which compresses the optic nerve head at the level of the lamina cribrosa, impairing axonal transport, particularly the retrograde axoplasmic flow, resulting in the accumulation of intracellular materials and subsequent swelling of the optic nerve head [6, 7, 8]. Fundoscopy traditionally assesses papilledema, but it is subjective and lacks quantitative accuracy [9]. Optical coherence tomography (OCT), by contrast, offers precise measurements of peripapillary retinal nerve fibre layer (pRNFL) thickness, which has been widely used in clinical practice for monitoring papilledema [9, 10, 11]. However, pRNFL thickness measurements can also be elevated in other conditions, such as optic neuritis, optic disc drusen (ODD), and retinal vascular diseases, making it more difficult to establish an accurate diagnosis [12, 13, 14].

An important parameter to distinguish papilledema from other optic disc oedema types is the “anterior displacement” of the optic disc (ADOD) and peripapillary tissues, which occurs due to the transmission of elevated intracranial pressure along the perioptic subarachnoid space [10, 15]. Increased pressure leads to specific mechanical changes, including axoplasmic stasis, pRNFL thickening and optic nerve head enlargement [10, 15]. However, indicators such as pRNFL thickness and optic disc elevation often require several weeks to reflect significant changes, posing a challenge in assessing early treatment response in papilledema [16, 17].

Multiple studies have investigated early treatment responses for papilledema within 1‐month intervals, focusing on diverse therapeutic approaches, including medical and surgical approaches [18, 19, 20, 21, 22]. While most studies did not specifically examine ADOD as a treatment marker, one study utilised geometric morphometrics to analyse the shape of the peripapillary retinal pigment epithelium/Bruch's membrane (ppRPE/BM) layer [15]. This approach provides a sophisticated method for assessing structural changes, though it remains underutilised in routine clinical evaluation. In this study, the group receiving clinical treatment demonstrated outcomes only in the long term, and the absence of a specified interval limits its applicability as an indicator of early treatment response.

Our study aims to address the knowledge gap by evaluating ADOD as both a diagnostic marker for papilledema and a potential biomarker for treatment response. Using advanced imaging techniques and quantitative analysis, we seek to identify structural changes in the peripapillary region that reflect therapeutic effects. This study aims to create a reliable, reproducible framework to support clinicians in monitoring treatment response more effectively and guiding timely interventions, ultimately redefining how early management of papilledema is approached.

## 2 Methods

This study is a retrospective, descriptive, observational, and comparative study involving participants diagnosed with papilledema secondary to IIH and cerebral venous sinus thrombosis (CVST) treated at the Hospital de Olhos Juiz de Fora—MG between January 2015 and February 2024. For controls, participants diagnosed with ODD were selected and matched by sex and age during the same period. ODD are characterised by the presence of hyaline bodies located anterior to the lamina cribrosa of the optic disc, with nodular shape and composed of mucopolysaccharides and mucoproteins, which may contain calcium deposits in their composition. This condition was chosen for its often bilateral nature, lack of short‐term visual impairment, and frequent misdiagnosis as papilledema, making it the most common cause of pseudo‐optic disc oedema. Additionally, on OCT examination, ODD is characterised by increased pRNFL thickness without ADOD and adjacent peripapillary structures, making them a more appropriate control group for comparisons with papilledema cases.

Participants with papilledema and ODD underwent a comprehensive ophthalmological evaluation, adhering to standardised acquisition and analysis protocols, including best‐corrected visual acuity (BCVA) assessment, ocular motility testing, pupil examination, anterior segment biomicroscopy, intraocular pressure measurement, fundoscopic examination and OCT imaging.

**FIGURE 1:**
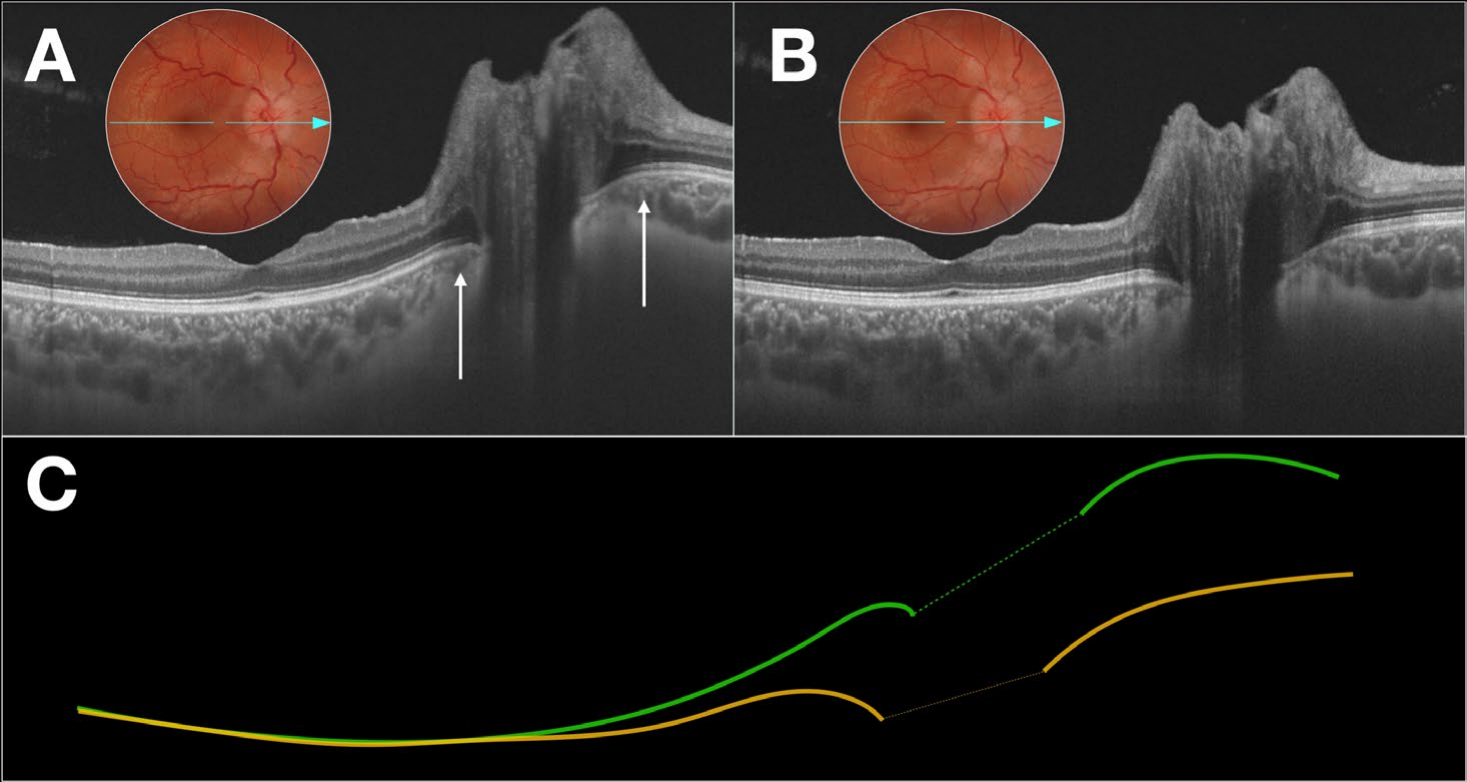
(A) High‐resolution B‐scan optical coherence tomography (OCT) (12 × 9 mm) of a patient with idiopathic intracranial hypertension at baseline, demonstrating optic disc oedema and anterior displacement of the optic disc (ADOD) (white arrows) at the time of diagnosis. (B) The same OCT scan obtained at the same position 10 days after the initiation of medical treatment, showing persistent optic disc elevation but a significant reduction in anterior displacement of the optic disc. A corresponding colour fundus photograph taken at the same time as the OCT scan is shown in both images. (C) Schematic representation of the retinal pigment epithelium (RPE) profiles from images (A—green line) and (B—yellow line) overlaid to illustrate the reduction in anteroposterior displacement of the optic disc. The dashed line in the center represents the Bruch's membrane opening, delineating the RPE interval.

To be included, participants were required to have undergone OCT examination with the acquisition of high‐resolution images of the optic disc and macular region. The device used was the DRI OCT‐1 Triton Topcon, a swept‐source OCT device providing high‐resolution cross‐sectional B‐scan images with a scan speed of 100 000 A‐scans/s and three‐dimensional volumetric images. The image acquisition protocol was the B‐scan line, an axial transverse image measuring 12 mm with a scan density of 1024 B‐scans, passing simultaneously through the optic disc and the center of the fovea (Figure 1). For participants with papilledema, images were analysed at the time of diagnosis (baseline, Figure 1A) and again 7–30 days after the initiation of treatment (Figure 1B). The standard initial treatment for most patients consisted of oral acetazolamide at a dosage of 1 g per day. In patients with CVST, anticoagulation therapy was administered in addition to the use of acetazolamide. The treatment was adjusted based on the initial response, which was evaluated through improvements in symptoms such as headaches, transient visual obscurations and changes in optical disc oedema as assessed by both fundus examination and OCT imaging. Additionally, in cases of IH associated with obesity, patients were advised to pursue weight loss alongside pharmacological treatment to aid in reducing IH and improving overall outcomes.

**FIGURE 2:**
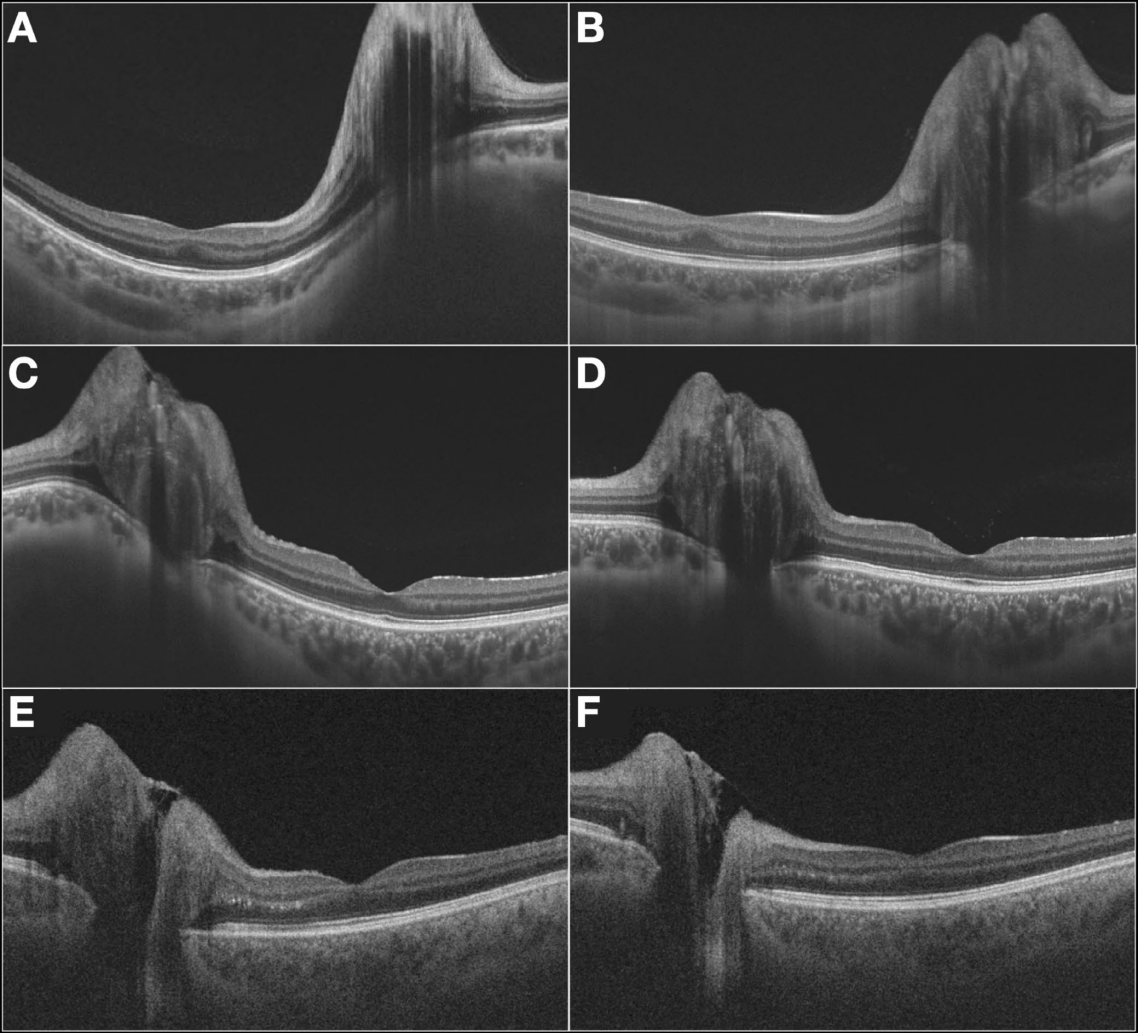
(A, B) High‐resolution B‐scan optical coherence tomography (OCT) (12 × 9 mm) of a patient with papilledema at baseline (A), demonstrating optic disc oedema and anterior displacement of the optic disc (ADOD). After medical treatment (B), there is complete resolution of ADOD, despite persistent optic disc elevation. (C, D) OCT scans of a second patient at baseline (C), showing optic disc oedema with ADOD. Following treatment (D), there is a significant reduction in ADOD, though some degree of anterior displacement persists. (E, F) OCT scans of a third patient with papilledema, but without ADOD at baseline (E) or after treatment (F).

Two independent and masked examiners for diagnosis analysed the transverse axial images acquired from participants with papilledema and ODD. The transverse axial images were analysed, totaling two images from each eye, one at baseline and one after treatment for the papilledema group, and at different times for the ODD group (Figure 2). The examiners were asked to indicate with “yes or no”: (1) the presence of optic disc elevation in the baseline images; (2) the presence of ADOD in the baseline images; (3) the presence of optic disc elevation in the post‐treatment images; (4) whether there was a reduction in optic disc elevation between images; (5) the presence of ADOD in the post‐treatment images; and (6) whether there was a reduction in ADOD between images. The examiners were blinded to the participants' diagnoses (papilledema or ODD), and the images were randomly selected for evaluation.

Participants eligible for this study were required to meet specific inclusion criteria tailored to ensure diagnostic accuracy and consistency. For individuals diagnosed with papilledema secondary to IIH, eligibility was based on a confirmed diagnosis established according to the criteria outlined above. Contrast‐enhanced cerebral magnetic resonance angiography for the venous phase was performed in all cases to distinguish IIH from CVST. Additionally, participants were required to have undergone a comprehensive ophthalmological examination, including OCT imaging, and to receive appropriate treatment with follow‐up until discharge. Discharge was defined as achieving control of IH and complete resolution of papilledema.

Specific inclusion criteria for participants with papilledema included the following: all participants must have undergone brain magnetic resonance imaging to confirm diagnosis. BCVA had to range between 20/400 and 20/20 in the eye being evaluated, with refractive errors restricted to less than five spherical diopters and three cylindrical diopters. Intraocular pressure (IOP) was required to be below 22 mmHg, and participants had to demonstrate sufficient cooperation to complete the ophthalmological and OCT examinations. Furthermore, individuals who had received previous treatment for papilledema were excluded from participation.

For the control group, which consisted of participants with ODD, eligibility was determined by a complete ophthalmological examination and OCT imaging. Participants in this group were required to have BCVA of 20/20 in both eyes, refractive errors less than five spherical diopters and three cylindrical diopters, and IOP below 22 mmHg. They also needed to be in good general health, free of systemic diseases and able to cooperate fully during the examinations.

Exclusion criteria applied to both groups were established to ensure the reliability of the findings. Participants were excluded if they had IH secondary to expansive intracranial processes or hydrocephalus, or if they had already begun treatment for papilledema prior to the first OCT imaging. Patients who required neurosurgical treatment, such as CSF shunt placement or optic nerve sheath fenestration, were also excluded. Additional exclusion criteria included the presence of eye diseases such as glaucoma, other optic neuropathies, or macular disorders; systemic conditions like systemic arterial hypertension or diabetes mellitus; and any media opacity, such as cataracts, that could interfere with the accuracy or completion of examinations. Participants who had undergone previous ocular surgeries, other than refractive surgery or uncomplicated cataract surgery performed more than 6 months prior, were also excluded.

The statistical analysis was performed to evaluate the diagnostic ability of transverse axial OCT images and pRNFL thickness measurements in identifying and monitoring papilledema. Interobserver and intraobserver variability in detecting ADOD and peripapillary tissues was assessed using Cohen's *κ* coefficient. Mean pRNFL thickness changes from baseline to post‐treatment were analysed for significance. The correlation between pRNFL thickness and ADOD was assessed using Spearman's correlation, while logistic regression determined the association between mean pRNFL thickness and ADOD likelihood at baseline and post‐treatment. ADOD presence and reductions were reported as proportions, with precise percentages for observer assessments.

## 3 Results

Eighty‐two eyes from 41 patients were included, with 25 (50 eyes) having papilledema and 16 (32 eyes) with ODD. The age distribution of cases and controls is shown in Table 1. Forty eyes with papilledema (80.0%) had BCVA of 20/20 at presentation, eight eyes (16.0%) had BCVA between 20/25 and 20/40, and two eyes (4.0%) had a BCVA between 20/200 and 20/400. There was no statistically significant difference between the BCVA of the papilledema eyes in the baseline and post‐treatment (*p* = 0.053). The majority of patients did not exhibit vision loss at the time of presentation.

**TABLE 1:** Baseline demographics of the papilledema and optic disc drusen groups.

Papilledema group		
Total eyes	50	
Total patients	25	
Age, years (mean, SD)	31.8	12.6
Female (*n*, %)	23	92%
Weight (kg; mean, SD)	82.8	12.9
Height (cm; mean, SD)	163.0	5.08
BMI (kg/m^2^; mean, SD)	31.4	4.6
Follow‐up interval (days; mean, SD)	15.8	7.1
Diagnosis—Idiopathic Intracranial Hypertension (*n*, %)	21	84%
Diagnosis—Central venous trombosis (*n*, %)	4	16%

Optic disc drusen control group		
Total eyes	32	
Total patients	16	
Age, years (mean, SD)	28.8	18.0
Female (*n*, %)	12	75%

Abbreviations: BMI, body mass index; cm, centimetres; kg, kilograms; kg/m^2^, kilograms/meters square; SD, standard deviation.

The ADOD was present in 50% of papilledema eyes at baseline, based on the mean prevalence reported by both observers (observer 1: 26 eyes, 52%; observer 2: 24 eyes, 48%), and in 32% post‐treatment (observer 1: 15 eyes, 30%; observer 2: 17 eyes, 34%). A decrease in the ADOD between images was noted in 17 cases (34.0%) for both observers. All the patients who exhibited ADOD, both at baseline and post‐treatment, also showed optic disc elevation for both observers. None of the eyes in the ODD group demonstrated ADOD at any time point. Table 2 summarises the results from the observers' evaluation.

**TABLE 2:** Summary of the masked observers' analysis, detailing baseline and post‐treatment assessments and reduction trends over time.

Papilledema group	Observer 1 (yes/no)	Observer 2 (yes/no)	Cohen's *κ* coefficient (Overall)
Presence of optic disc elevation (baseline)	50/0	49/1	0.97^a^
Presence of ADOD (baseline)	26/24	24/26	0.84
Presence of optic disc elevation (post‐treatment)	50/0	49/1	0.97^a^
Presence of ADOD (post‐treatment)	15/35	17/33	0.725
Reduction in optic disc elevation between images	19/31	26/24	0.643
Reduction in ADOD between images	17/33	17/33	0.643

Optic disc drusen group	Observer 1 (yes/no)	Observer 2 (yes/no)
Presence of optic disc elevation (first image)	24/26	24/26^a^
Presence of ADOD (first image)	0/50	0/50
Presence of optic disc elevation (second image)	24/26	23/27
Presence of ADOD (second image)	1/50	0/50
Reduction in optic disc elevation between images	0/50	0/50
Reduction in ADOD between images	0/50	0/50

Abbreviations: ADOD, anterior displacement of the optic disc; ODD, optic disc drusen.

^a^ Calculated with Gwet's AC1 coefficient.

At baseline, optic disc elevation was present in all papilledema eyes assessed by observer 1 (50 eyes, 100%) and in 49 eyes (98%) for observer 2. Post‐treatment, the prevalence of optic disc elevation remained unchanged for both observers. However, despite this stability in prevalence, a reduction in the intensity of optic disc elevation was observed in 19 eyes (38%) for observer 1 and in 26 eyes (52%) for observer 2.

The reproducibility and consistency of the method were assessed using Cohen's *κ* coefficient. Due to the high prevalence of optic disc elevation at both time points, the *κ* values for its presence at baseline and post‐treatment were 0, despite a 98% agreement between observers. To correct for prevalence and bias we recalculated this with Gwet's AC1 coefficient, which provided a value of 0.97, indicating strong agreement. For other parameters, the *κ* values were as follows: presence of ADOD at baseline, 0.84 (excellent); presence of ADOD post‐treatment, 0.725 (good); reduction in optic disc elevation between images, 0.643 (moderate); and reduction in ADOD between images, 0.643 (moderate). Considering the overall assessment, the *κ* coefficient for all evaluations was 0.799, indicating substantial agreement.

**FIGURE 3:**
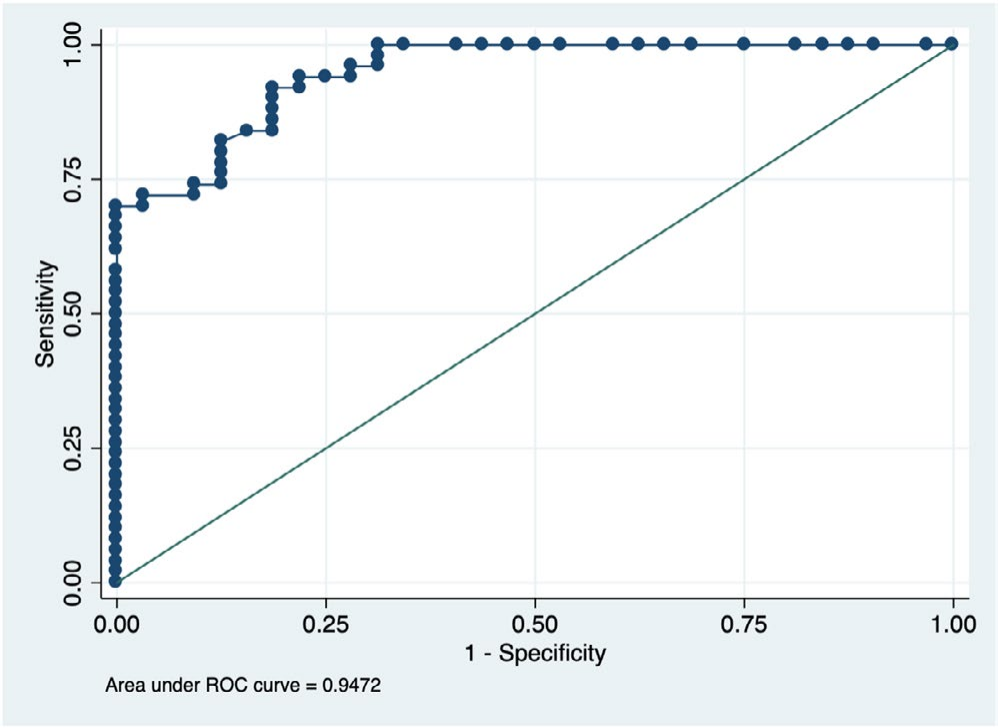
Receiver operating characteristic (ROC) curve depicting the diagnostic performance of peripapillary retinal nerve fibre layer thickness in differentiating papilledema from optic disc drusen. The curve illustrates the sensitivity and specificity trade‐off, with the area under the curve (AUC) value of 0.9472.

To assess the diagnostic performance of pRNFL thickness in distinguishing eyes with papilledema from ODD (control group), a logistic regression analysis was performed, followed by the construction of a receiver operating characteristic (ROC) curve. The model demonstrated excellent discriminative ability, with an area under the curve (AUC) of 0.9472, indicating a high level of accuracy in differentiating between the two conditions. The ROC curve is presented in Figure 3.

The mean pRNFL thickness measurements in the papilledema group showed a significant decrease when comparing baseline measurements to post‐treatment values (305.36 ± 140.18 mm compared to 245.26 ± 94.45 mm, *p* < 0.001).

**FIGURE 4:**
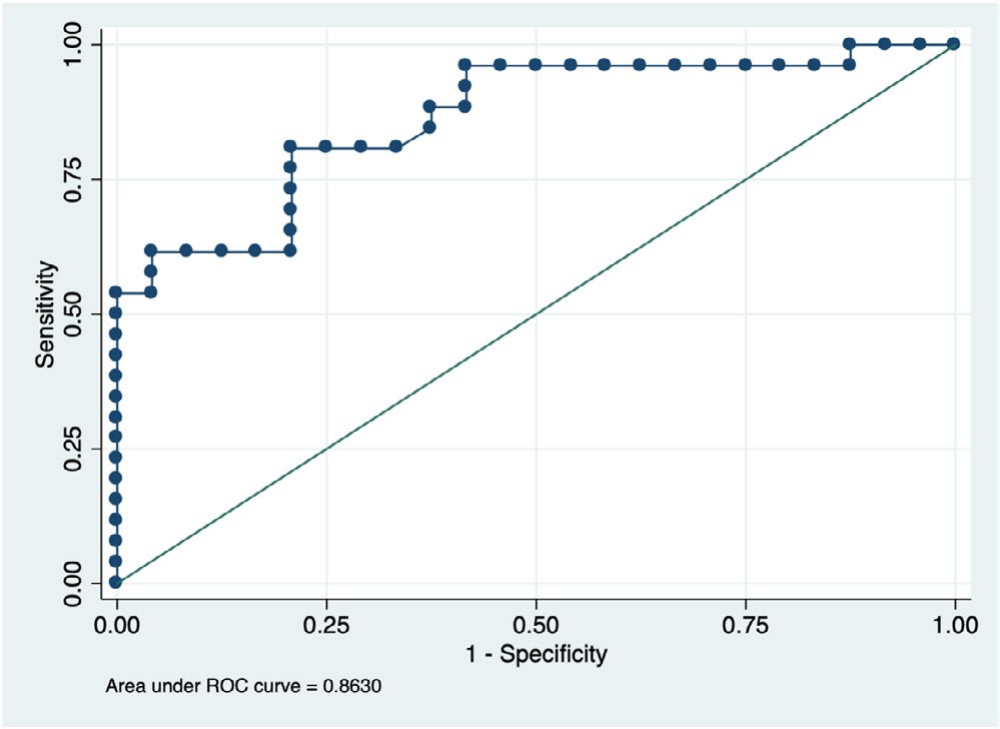
Receiver operating characteristic (ROC) curve illustrating the association between peripapillary retinal nerve fibre layer (pRNFL) thickness and anterior displacement of the optic disc. Logistic regression analysis demonstrated an area under the curve (AUC) of 0.8630. The optimal pRNFL cutoff for detecting anterior displacement was ≥ 264 μm, with a sensitivity of 80.77% and a specificity of 79.17% (Youden index = 0.5994).

Logistic regression analysis demonstrated that the baseline mean pRNFL thickness was significantly associated with ADOD (odds ratio [OR] = 1.016, *p* = 0.001), and that the mean RNFL thickness post‐treatment was also associated with ADOD (OR = 1.012, *p* = 0.004). The model demonstrated good discriminative ability, with an AUC of 0.8630, presented in Figure 4. The best cutoff for pRNFL indicating ADOD was ≥ 264 μm, with 80.77% sensitivity and 79.17% specificity (Youden index of 0.5994), while a higher threshold of ≥ 383 μm yielded 100% specificity (Youden index of 0.5385).

Spearman correlation revealed that a positive moderate correlation was found between ADOD and average RNFL thickness measurements of patients with papilledema in the baseline (*r* = 0.628 and *p* < 0.001), but this was poor in the post‐treatment (*r* = 0.413 and *p* = 0.003).

A mixed‐effects model accounted for inter‐eye correlation when assessing the association of body weight, height, and BMI with baseline pRNFL thickness but found no significant relationship (Wald *χ* ^2^ (3) = 1.38, *p* = 0.7111). None of the predictors—weight (*β* = 5.72, *p* = 0.514), height (*β* = −5.06, *p* = 0.631) or BMI (*β* = −20.80, *p* = 0.389)—showed significant effects. Similarly, a GEE model with a binomial link function found no association between BMI and ADOD (Wald *χ* ^2^(1) = 0.47, *p* = 0.4925; *β* = −0.054, *p* = 0.493).

## 4 Discussion

Our study suggests that ADOD may serve as an important biomarker for differentiating papilledema from ODD. Importantly, ADOD was not observed in any eyes with ODD, reinforcing its potential diagnostic specificity. When assessed alongside pRNFL thickness measurements, ADOD has the potential to enhance the diagnostic evaluation and monitoring of papilledema. Moreover, ADOD may represent a more sensitive or specific structural indicator of response to treatment in papilledema secondary to IIH and CVST. In all studied cases, the reduction in ADOD occurred despite the persistence of increased pRNFL thickness measurements. Additionally, using the same OCT images, we successfully detected ADOD with strong reproducibility and consistency between the two observers. Baseline and post‐treatment ADOD showed a significant correlation with the severity of optic disc oedema. Notably, greater mean pRNFL thickness was associated with more pronounced optic disc oedema and a higher likelihood of ADOD both before and after treatment. The treatment of papilledema effectively reduced the ADOD, even before a complete reduction in pRNFL thickness, highlighting the utility of OCT imaging for both diagnostic and therapeutic monitoring in the management of papilledema.

The significant reduction in pRNFL thickness observed after treatment reflects the expected decompression of retinal nerve fibres following the reduction of intracranial pressure. This finding is consistent with previous studies suggesting that pRNFL thickness serves as an indirect marker of optic disc oedema severity and treatment response [9, 10, 11]. While pRNFL thickness decreases gradually over weeks, ADOD reduction may precede substantial structural normalisation, reinforcing its potential as a biomarker of improvement.

The extent of ADOD appears linked to pre‐treatment oedema burden, as reflected by baseline pRNFL thickness. A pRNFL threshold of ≥ 264 μm provided an optimal balance of sensitivity (80.77%) and specificity (79.17%), whereas a higher threshold of ≥ 383 μm showed 100% specificity. However, this relationship is neither linear nor threshold‐dependent, suggesting modulation by individual biomechanical factors such as scleral rigidity, lamina cribrosa compliance, and peripapillary connective tissue properties. Although higher BMI has been linked to increased IIH risk [23], our findings suggest it does not influence pRNFL thickness or ADOD in IIH patients. However, interpretation should be cautious, as all patients were obese, improved with treatment and weight loss, and the sample size was limited.

**FIGURE 5:**
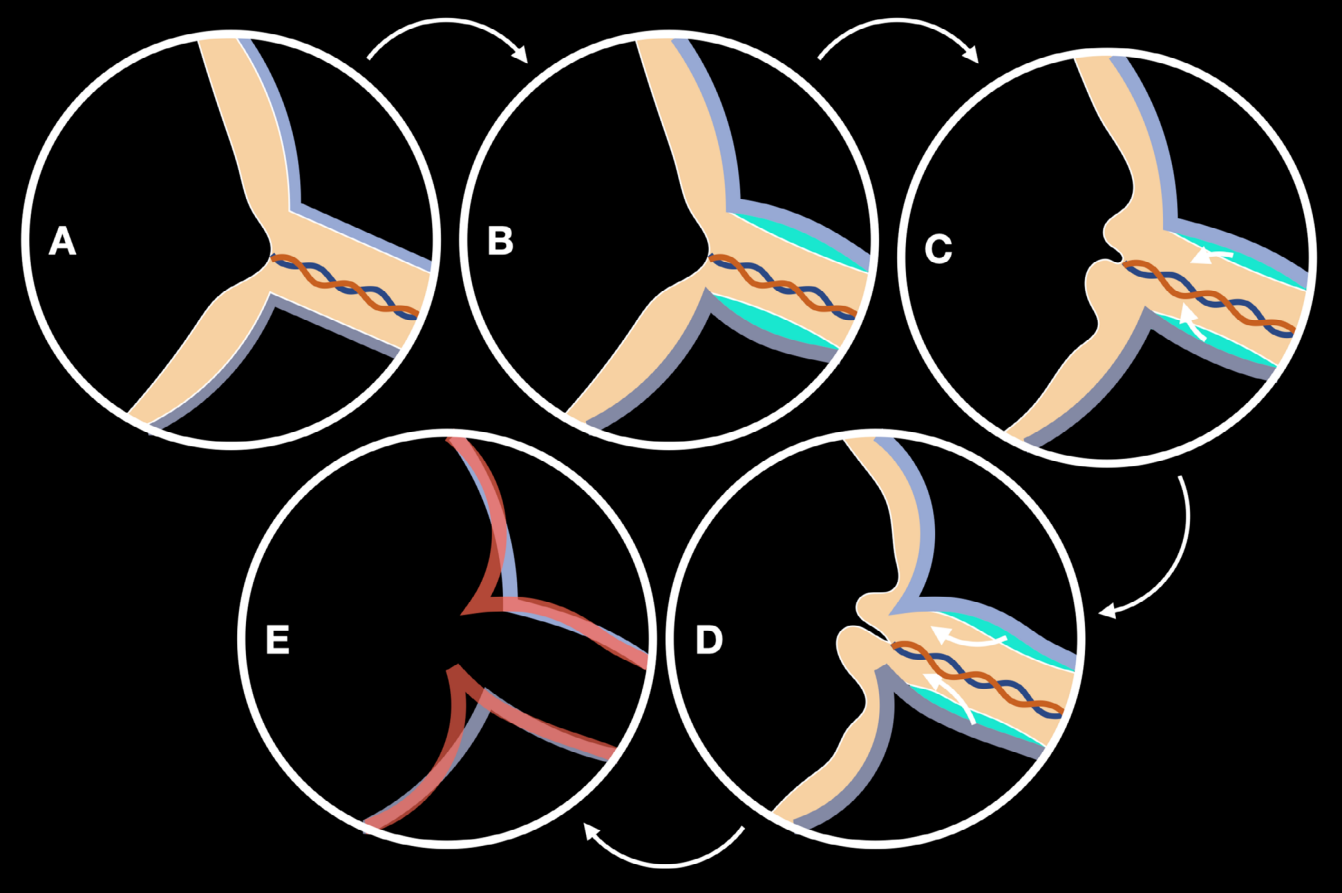
Schematic representation illustrating the progressive anterior displacement of the optic disc in papilledema. (A) Depiction of the posterior globe anatomy highlights the optic nerve's distal portion and its insertion into the eye under normal conditions. (B) Increased intracranial pressure leads to cerebrospinal fluid accumulation (in blue) within the optic nerve sheath, resulting in optic nerve swelling. (C) This swelling, driven by fluid influx (white arrows), contributes to the bulging of the optic disc. (D) As intracranial pressure continues to rise, peripapillary tissues are progressively pushed forward (white arrows), leading to further anterior displacement. (E) Overlay schematic comparing the retinal pigment epithelium profiles from images (C—in purple) and (D—anterior displacement, in red) to illustrate the projection of the optic disc toward the vitreous.

Papilledema results from optic nerve compression behind the lamina cribrosa due to IH, obstructing axoplasmic flow and causing axonal swelling around the optic nerve head (Figure 5) [24, 25]. The extent of pRNFL thickening can be precisely assessed using spectral‐domain OCT by measuring its average thickness around the optic disc [26, 27, 28, 29]. Previous research, including Scott et al. [9], validated mean pRNFL thickness as a reliable indicator of optic disc swelling severity, correlating with Frisen‐graded optic disc photographs. Although Sibony et al. [10] consider pRNFL thickness a reliable marker of papilledema severity, they acknowledge that peripapillary subsurface shape analysis complements IH evaluation.

Sibony and Kupersmith reported that the ADOD depends on intracranial pressure rather than optic disc oedema and is influenced by scleral biomechanics. For instance, patients with ischemic disc oedema, such as in nonarteritic anterior ischemic optic neuropathy (NAION), show no deformation, reinforcing ADOD as a more specific biomarker for papilledema [10].

Previous studies have shown that mean pRNFL thickness decreases after the intervention, with a small decrease after a lumbar puncture and larger drops after a CSF shunt or medical treatment [15, 16]. Similarly, early improvements in papilledema have been documented across various treatment approaches, with notable changes occurring within the first month. For instance, Elnahry et al. [22] reported improved peripapillary microperimetry thresholds in IIH patients treated with acetazolamide and weight reduction. In a surgical context, Alsuhaibani et al. [21] noted rapid reductions in papilledema grading as early as 2 weeks post‐optic nerve sheath fenestration, while Koktekir et al. [20] observed substantial pRNFL thickness reduction within the first week following CSF shunting or ventriculostomy, which continued to decrease over the subsequent month. However, none of these studies addressed ADOD. Additionally, time to full papilledema resolution can take approximately 6 months, according to Liu et al. [19].

Although Sibony et al. [15] analysed anterior shape deformation using geometric morphometrics to examine the ppRPE/BM layer on OCT images, this technique involves complex software applications and has yet to be broadly implemented in clinical practice. Our study builds on this work by assessing ADOD as a more accessible and immediate indicator of treatment response.

In our study, we focused specifically on the ADOD of the peripapillary region as a structural biomarker for assessing treatment response in papilledema secondary to IIH and CVST. Our results demonstrated that ADOD was present in 50% of the eyes with papilledema at baseline and decreased significantly to 32% within the first month of medical treatment. This ADOD strongly correlated with the severity of optic disc oedema, as measured by pRNFL thickness, both pre‐ and post‐treatment. Importantly, these findings indicate that ADOD may serve as a valuable biomarker of therapeutic effectiveness, offering a practical method to evaluate initial treatment outcomes in a shorter time frame.

These early insights are particularly valuable as they suggest a new approach to follow‐up in this patient group. In clinical practice, follow‐up after initiating treatment is often scheduled within 1–3 months, although intervals are individualised and may be shorter in more severe cases. Our findings suggest that a closer follow‐up interval, between 1 and 4 weeks, can yield meaningful information on the initial therapeutic response, providing clinicians with an earlier understanding of patient progress. It is important to note that our cohort only includes patients undergoing medical treatment, without surgical intervention.

This study has several limitations. First, its retrospective design introduces biases, including reliance on pre‐existing data and variability in follow‐up intervals. Second, the small sample size limits statistical power and generalisability. Third, while OCT analysis by two experienced ophthalmologists showed good interobserver reproducibility, its qualitative nature may limit applicability to less experienced observers. Inconsistent follow‐up intervals were mitigated by excluding patients with intervals exceeding 30 days to focus on early treatment response.

In conclusion, our study shows that OCT effectively differentiates papilledema from ODD and assesses ADOD and optic disc oedema. The correlation between pRNFL thickness and optic disc oedema, pre‐ and post‐treatment, underscores OCT's diagnostic value. Our findings confirm that pRNFL thickness reduction and ADOD improvement post‐treatment are valuable biomarkers for papilledema recovery. Integrating these OCT metrics provides a robust framework for diagnosing and managing papilledema.

## Ethics Statement

The Research Ethics Committee of the Federal University of Juiz de Fora reviewed and approved this study protocol. The participants provided informed consent, and the confidentiality of the data collected during the survey is maintained by the authors.

## Conflicts of Interest

The authors declare no conflicts of interest.

## Data Availability

The authors are responsible for the data in the manuscript and assure full availability of the study material.
